# New Approach to Develop Optimized Sunscreens that Enable Cutaneous Vitamin D Formation with Minimal Erythema Risk

**DOI:** 10.1371/journal.pone.0145509

**Published:** 2016-01-29

**Authors:** Dieter Kockott, Bernd Herzog, Jörg Reichrath, Kevin Keane, Michael F. Holick

**Affiliations:** 1 UV-Technik, Hanau, Germany; 2 BASF Grenzach GmbH, Grenzach-Wyhlen, Germany; 3 Universitätsklinikum des Saarlandes, Homburg/Saar, Germany; 4 Endocrinology, Diabetes and Nutrition, Department of Medicine, Boston University Medical Center, Boston, Massachusetts, United States of America; Medical University of Gdańsk, POLAND

## Abstract

Sunscreens protect the skin against erythemal radiation (E_er_). But at the same time they reduce the effective radiation dose (E_VD_) responsible for the formation of previtamin D in the skin. The paper describes a calculation method for optimizing the ratio E_VD_/E_er_ behind sunscreens e.g. with SPF 5, 15 and 30 respectively. Taking into account that a majority of people in industrialized countries suffer from a shortage in vitamin D even in summer time, the ratio E_vd_/E_er_ is a new and important criterion for the quality of sunscreens. Furthermore the exposure time t_vd_ needed per day for forming the equivalent of the recommended amount of 2000 IU of vitamin D per day for skin type 2 is estimated when sunscreens with different filter compositions are used. *In vitro* experiments show a significant increase of the conversion of 7-dehydrocholesterol (7-DHC) to previtamin D when exposed to artificial solar radiation behind an experimental sunscreen optimized for previtamin D production compared to a commercial sunscreen having the same SPF.

## Introduction

Solar ultraviolet radiation is responsible for erythema and represents the major environmental risk factor that contributes to the pathogenesis of non-melanoma skin cancer [1]. Consequently, strict UV-protection by using sunscreens and by other measures represents a fundamental part of many skin cancer prevention campaigns [1–4]. However, we are in a dilemma, that strict UV-protection promotes vitamin D deficiency, that is associated with an increased risk for and an unfavourable outcome of many diseases [3,4]. Under most living conditions, at least 80% of the human body’s need in vitamin D has to be synthesized in the skin by the action of solar or artificial UV-B radiation, only a small amount of vitamin D is taken in by the diet [3,4]. Increasing evidence now supports the concept that vitamin D deficiency has enormous consequences for human health [3,4]. Recent assessments of the 25-hydroxyvitamin D (25(OH)D) status concluded that about 40% of children and adults in the United States, Canada, Europe, Asia, India, South America and Australia are vitamin D deficient [3,4]. Besides musculo-skeletal consequences, that include rickets in children, osteomalacia, fractures, muscle weakness and falls in older adults, a growing number of other acute and chronic illnesses including many types of cancer (breast, colon etc.), autoimmune diseases (e.g., type 1 diabetes, multiple sclerosis and rheumatoid arthritis), infectious diseases, neurocognitive dysfunction including Alzheimer’s disease, type 2 diabetes and cardiovascular disease have now been linked by epidemiological, observational and experimental studies associated with vitamin D deficiency [3,4]. As a result of this dilemma, there is at present an enormous need to develop optimized sunscreens that enable maximum cutaneous vitamin D formation with a given quantity of erythema protection described by the sun protection factor (SPF).

The sunscreen industry promotes sunscreens with so called broad band filter approaching more or less uniform spectral absorbance across the UV spectrum. This concept is useful for actions with unknown action spectra. In this paper we focus only on both erythema and vitamin D with corresponding action spectra. For this application the use of erythema effective irradiance and vitamin D effective irradiance is more specific.

The paper describes a new promising strategy to reach the goal of vitamin D optimized sunscreens that is based on a theoretical approach. With the spectral solar irradiance E_λ_(λ), the spectral transmittance T(λ) of a given sunscreen and both, the action spectrum of erythema s_er_(λ) and the action spectrum of previtamin D formation s_vd_(λ) in the skin, it is possible to calculate the effective irradiance E_er_ = ∫ E_λ_(λ) s_er_(λ) T(λ) d λ for erythema as well as the effective irradiance E_vd_ = ∫ E_λ_ (λ) s_vd_(λ) T(λ) d λ for previtamin D formation in skin covered by a sunscreen. Sunscreens reduce necessarily both E_er_ and E_vd_ on skin. But by choosing the right filter composition the ratio E_vd_/E_er_ can be optimized. The higher the ratio, the better is the sunscreen concerning the previtamin D production for a given protection against E_er_.

The exposure time needed per day for forming the recommended amount of vitamin D depends on season, time of day, latitude and degree of skin pigmentation [4]. The topical application of a sunscreen with an SPF of 15 was reported to reduce the production of previtamin D in the skin by approximately 95% [5]. UV exposure of the whole human body by 1 MED for skin type 2 has been estimated to correspond to an oral intake of approximately 10000–20000 international units (IU) of vitamin D [4]. Although it has to be noted that there is still an ongoing debate concerning the daily requirement of vitamin D, it has been recommended by the Endocrine Practice Guidelines that adults need at least 1500–2000 IU vitamin D per day [6]. Thus, when knowing the spectral transmittance T(λ) of a sunscreen film on human skin, it is possible to estimate the time it would take in the presence of a sunscreen to form the amount of vitamin D needed per day.

**Fig 1:**
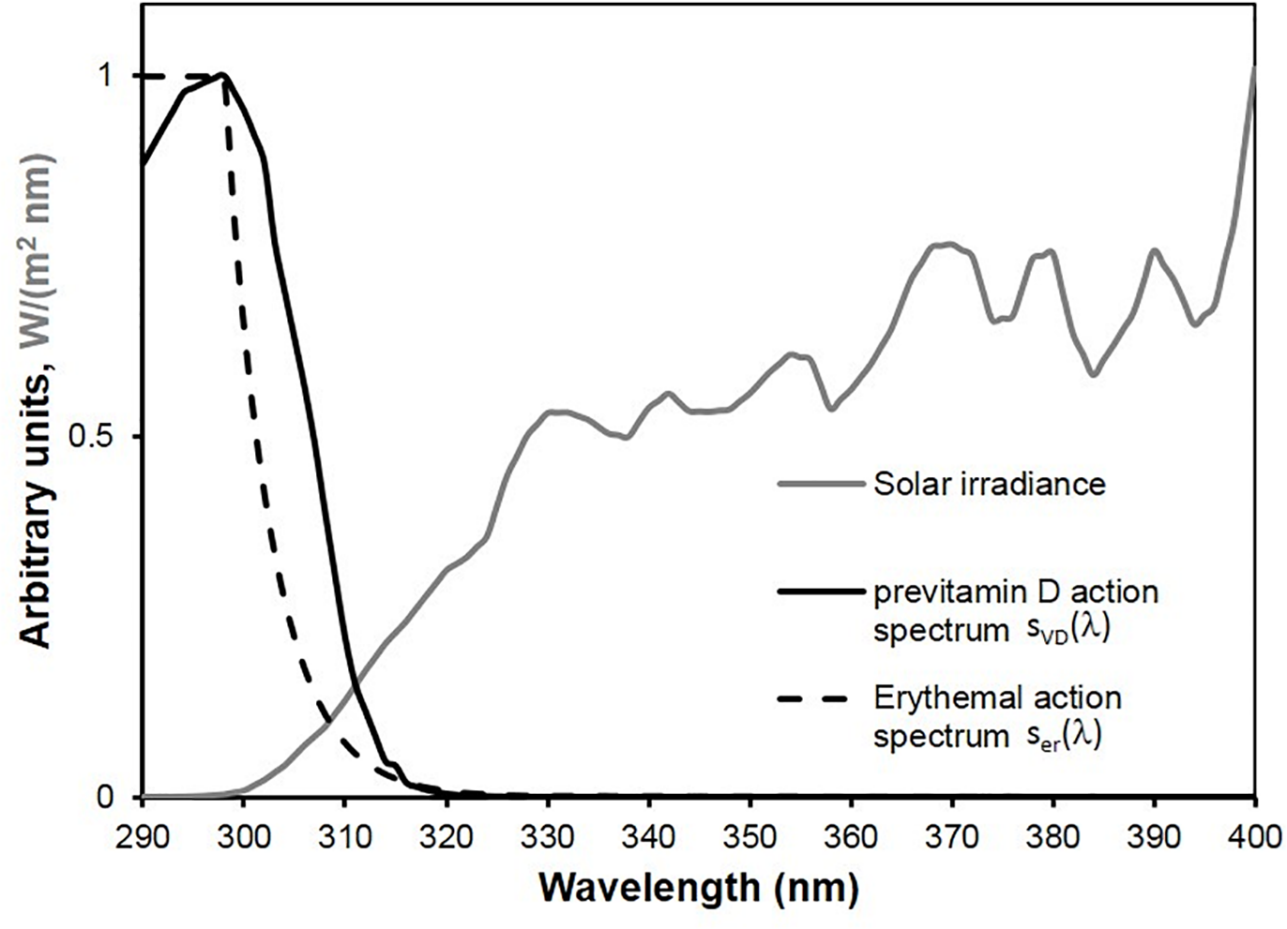
Action spectra for erythema and for previtamin D formation in human skin and standard solar spectral irradiance at the surface of the earth.

In this work different types of sunscreens are compared in terms of their protection against erythema effective irradiance and their ability to allow the formation of previtamin D. The action spectra for erythema s_er_(λ) [7] and for previtamin D formation in human skin s_VD_(λ) [8] and a standard spectrum of solar irradiance [9] are shown in Fig 1. Even if there are some scientific discussions on the action spectrum for previtamin D formation in human skin we used the internationally accepted s_VD_(λ) as given by CIE [8].

**Fig 2:**
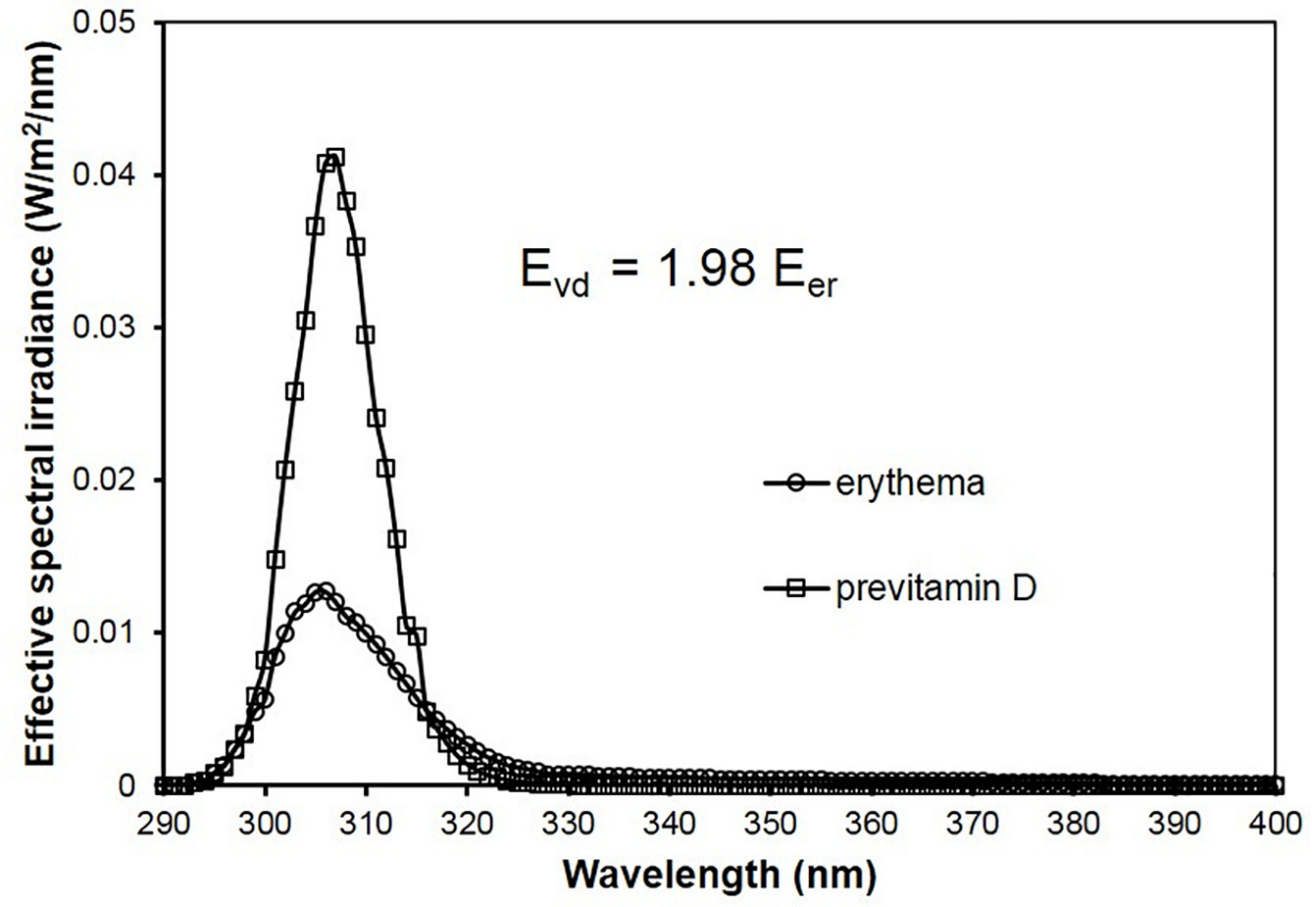
Effective spectral irradiances for both erythema and previtamin D formation when using standard spectrum of solar radiation COLIPA without sunscreen.

For a given spectral irradiance E_λ_ (λ) of a light source the effective irradiance E_er_ for erythema is defined by E_er_ = ∫ E_λ_ (λ) s_er_(λ) d λ and the effective irradiance E_VD_ for previtamin D formation is defined by E_VD_ = ∫ E_λ_ (λ) s_VD_(λ) d λ. The curves in Fig 2 represent the spectral effective irradiances for erythema E_λ_ (λ) s_er_(λ) and for previtamin D formation E_λ_ (λ) s_VD_(λ) respectively if E_λ_ (λ) is the standard spectrum of solar radiation defined by COLIPA [9] (COmite de LIasion des associations europeennes de l’industrie de la PArfumerie, des products cosmetiques et de toilette), today named Cosmetics Europe.

The calculation programme works as well for other spectral irradiances of solar radiation. But the differences are small because we focus on the ratio of previtamin D effective irradiance E_VD_ and erythema-effective irradiance E_er_. The areas below the two curves in Fig 2 represent E_VD_ and E_er_ respectively. The ratio E_vd_/E_er_ is about 2. With other words: The previtamin D-effective irradiance of standard solar radiation is about twice the erythema effective irradiance.

## Materials and Methods

For calculation of the spectral transmittance T(λ) of sunscreens with given filter compositions, a simulation tool www.basf.com/sunscreen-simulator was used and is available to the public [10,11,12]. The tool is based on the following elements: a database with UV spectra of the relevant UV filters (given as decadic molar extinction coefficients), a mathematical description of the irregularity profile of the sunscreen film on the skin, consideration of changes in UV filter concentration due to photoinstability, and consideration of formulation influences like the distribution of the UV filters in the oil and water phase of an emulsion [13]. All calculated results are related to an application amount of 2 mg/cm^2^.

Examples of filter compositions were chosen to achieve sun protection factors (abbreviated as SPF) of 6, 15, and 30. According to a recommendation of the European Commission (EC), an SPF of 6 belongs to the class of “low protection”, an SPF of 15 to the “medium protection” and an SPF of 30 to the “high protection” class. For each SPF four different types of spectral performance were constructed, with weak, intermediate, strong protection in the UV-A range and vitamin D optimized protection. The intermediate protection in the UV-A range means that the EC recommendation [13], which says that the ratio of UVA-PF/SPF should at least be 1/3 and the critical wavelength CW [14], at least 370 nm is fulfilled. For the case of low UV-A protection, the UVA-PF/SPF ratio is significantly lower, for that one of high UV-A protection or vitamin D optimized protection, it is far higher than 1/3.

In the following, abbreviations for the UV filter names, based on the international nomenclature of cosmetic ingredients (INCI) will be used: Ethylhexylmethoxy cinnamate (EHMC), octocrylene (OCR), phenyl benzimidazole sulfonic acid (PBSA), ethylhexyl triazone (EHT), titanium dioxide (TiO_2_), butyl methoxy dibenzoyl methane (BMDBM), diethylamino hydroxybenzoyl hexyl benzoate (DHHB), methylene bis-benzotriazolyl tetramethyl butylphenol (MBBT), and bis-ethylhexyloxy methoxyphenyl triazine (BEMT).

To confirm that these calculated theoretical values had some physiologic meaning i.e. could result in a sunscreen designed with ingredients that had UV absorption properties that would increase the UV-induced production of previtamin D3 an *in vitro* study was carried out. An array of fluorescent lamps with high ultraviolet output simulating UV solar radiation was used to irradiate borosilicate ampoules containing the previtamin D precursor 7-dehydrocholesterol (7-DHC). The ampoules were placed beneath a flat layer of polyethylene food wrap transmissive at UV wavelengths to which was applied a uniform amount of 2 mg/cm² either SPF-15 Solar D (UV filter composition: 1.9% octocrylene, 1% homomenthyl salicylate, 1% ethylhexyl salicylate, 3% avobenzone, 7.3% Diethylamino hydroxybenzoyl hexyl benzoate, and 0.9% diethylhexyl syingylidenemalonate) or SPF-15 commercial sunscreen Neutrogena, one of the most popular sunscreens used in the US (UV filter composition: 5% octisalate, 7.5% octinoxate, and 3% oxybenzone), or control (no sunscreen). The ampoules were placed on a piece of black paper to prevent re-irradiation by reflection with a flat cold pack beneath the paper to stabilize temperature. Six ampoules were irradiated at a time, for 5, 10, 15, 20, 25, or 30 minutes. Ampoules contents were analyzed after irradiation by high performance liquid chromatography (HPLC). For further experimental details see ref. [15].

## Results and Discussion

Table 1 shows the compositions as well as the calculated values of SPF, E_vd_/E_er_ ratio, UVA-PF, UVA-PF/SPF-ratio and critical wavelength CW [14] for four SPF 6 sunscreens, as explained above.

**Table 1:** UV filter compositions for SPF 6 with different levels of E_vd_/E_er_ ratio and UV-A protection.

Type	Filter Composition	SPF	E_vd_/E_er_	UVA-PF	UVA-PF/SPF	CW [nm]
	1% EHT 3% EHMC	**7.3**	**1.17**	1.3	0.18	335
2	0.5% BEMT, 0.8% DHHB, 2.5% EHMC	**7.4**	**1.75**	3.4	0.46	370
3	0.6% EHMC, 1% MBBT, 1% BEMT, 1% DHHB	**7.2**	**1.94**	7.1	0.99	377
4	7,5% DHHB, 3% BMDBM	**7.1**	**2.17**	16.4	2.32	379

**Fig 3:**
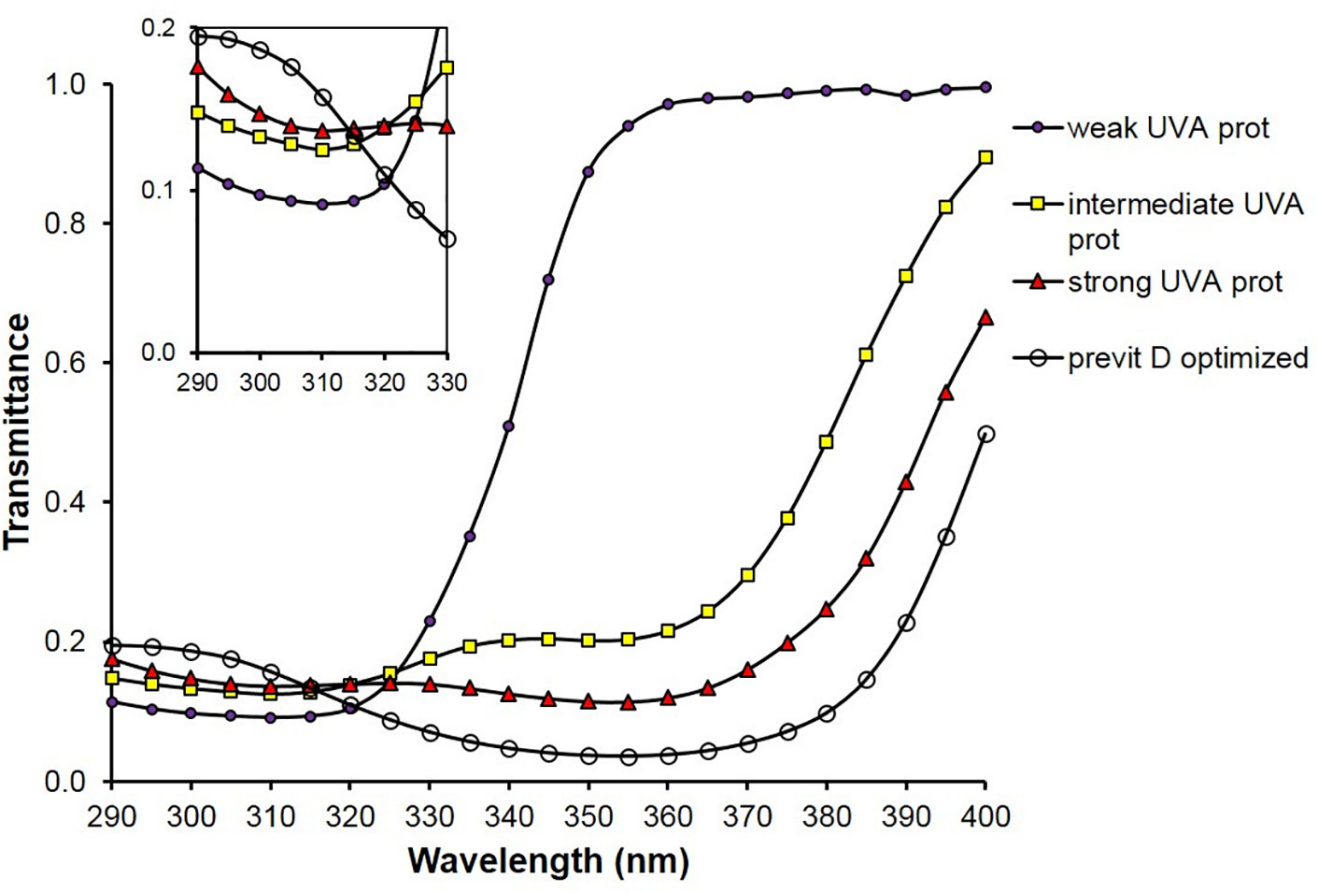
Spectra of simulated UV transmittances of four SPF 6 sunscreens (compositions in Table 1) with weak, intermediate, strong UV-A and previtamin D optimized protective properties; small graph: transmittance scale enlarged in the spectral range from 290 to 330 nm.

Fig 3 shows the simulated spectral transmittances of sunscreen films referring to the four different types of UV filter compositions leading to SPF 6.

Table 2 shows the compositions as well as the calculated values of SPF, E_vd_/E_er_ ratio, UVA-PF, UVA-PF/SPF-ratio and critical wavelength CW [14] for four SPF 15 sunscreens.

**Table 2:** UV filter compositions for SPF 15 with different levels of E_vd_/E_er_ ratio and UV-A protection.

Type	Filter Composition	SPF	E_vd_/E_er_	UVA-PF	UVA-PF/SPF	CW [nm]
1	2% PBSA, 3.5% EHT, 6% EHMC	**15.5**	**0.57**	1.4	0.09	333
2	1.5% DHHB, 2% BEMT, 4.3% EHMC	**15.6**	**1.71**	6.4	0.41	370
3	2% MBBT, 2% BEMT, 2% BMDBM, 2.5% OCR	**15.7**	**1.91**	15.6	0.99	378
4	3% BMDBM, 3.1% MBBT, 10% DHHB	**15.5**	**2.11**	30.2	1.95	380

**Fig 4:**
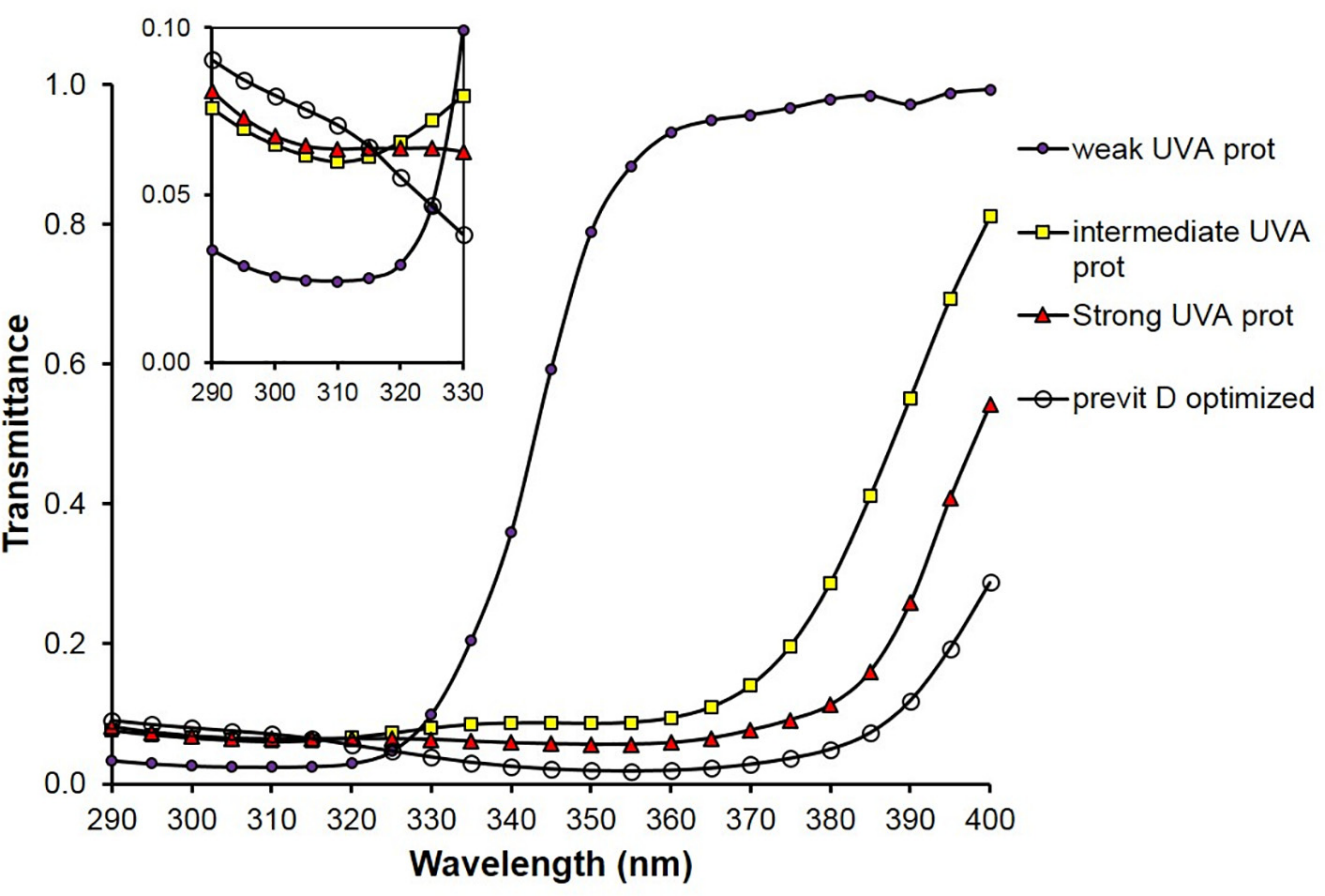
Spectra of simulated UV transmittances of four SPF 15 sunscreens (compositions in Table 2) with weak, intermediate, strong UV-A and previtamin D optimized protective properties; small graph: transmittance scale enlarged in the spectral range from 290 to 330 nm.

In Fig 4, the simulated spectral transmittances of sunscreen films referring to the four different types of UV filter compositions leading to SPF 15 are depicted.

Table 3 shows the compositions and the calculated values of SPF, E_VD_/E_er_ ratio, UVA-PF, UVA-PF/SPF ratio and critical wavelength CW [14] for four SPF 30 sunscreens.

**Table 3:** UV filter compositions for SPF 30 with different levels of E_vd_/E_er_ ratio and UV-A protection.

Type	Filter Composition	SPF	E_vd_/E_er_	UVA-PF	UVA-PF/SPF	CW [nm]
1	3% PBSA, 3% EHT, 3.5% TiO2, 10% EHMC	**33.1**	**0.70**	2.9	0.09	353
2	2% MBBT, 2.5% BEMT, 3% DHHB, 8% EHMC	**33.2**	**1.68**	2.3	0.37	374
3	2.2% PBSA, 3% BEMT, 4.5% MBBT, 4.5% DHHB	**33.2**	**1.84**	3.5	1.01	378
4	1.9% BEMT, 3% BMDBM, 7.4% MBBT, 10% DHHB	**33.1**	**2.00**	49.5	1.50	380

**Fig 5:**
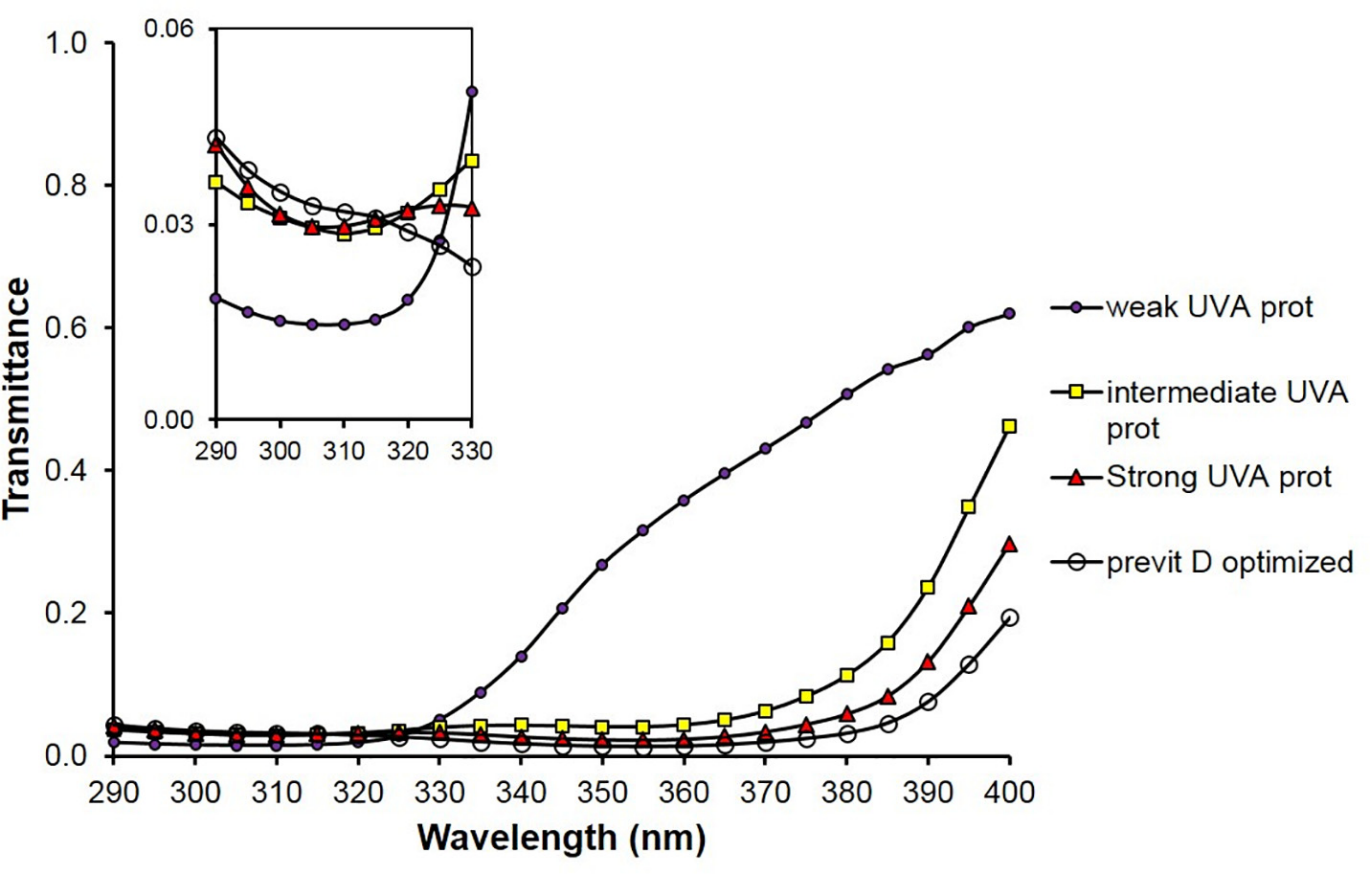
Spectra of simulated UV transmittances of four SPF 30 sunscreens (compositions in Table 3) with weak, intermediate, strong UV-A and previtamin D optimized protective properties; small graph: transmittance scale enlarged in the spectral range from 290 to 330 nm.

Fig 5 shows the simulated spectral transmittances of sunscreen films referring to the four different types of UV filter compositions leading to SPF 30.

Tables 1–3 demonstrate that the ratio E_VD_/E_er_ can be optimized to the value of about 2 which is typical for specified solar radiation (see Fig 2) even if the SPF of the sunscreens remains the same. That means the transmittance of sunscreens for previtamin D-effective radiation is optimized at the same level of protection against erythema-effective radiation. The corresponding Figs 3–5 show that those combinations of UV-absorbers are previtamin D optimized which have a relative high transmittance in the short UV range combined with a low transmittance in the long UV range.

For practical applications of sunscreens not only the ratio E_VD_/E_er_ is of importance, but also the exposure time t_VD_ needed to get the recommended amount of vitamin D/day. Even a sunscreen having a ratio E_VD_/E_er_ of about 2 doesn’t make sense in practice if the exposure time t_VD_ lasts e.g. 6 hours/day or more. The following estimate answers this question. It is known from scientific literature that whole body exposure of 1 MED = 250 J/m^2^ erythemal radiant exposure for skin type 2 is equivalent to an oral intake of about 10000–20000 IU Vitamin D [4]. For maximum solar input (standard sun COLIPA in zenith, cloudless sky) it takes about 20 minutes to get 1 MED. The recommended intake of vitamin D for adults ranges from 600 up to 2000 IU/day [5]. Thus to obtain 2000 IU/day equivalent the exposure time would be 1/5 x 20 min = 4 minutes when the unprotected skin is exposed to the above mentioned maximum solar input. Sunscreens attenuate the previtamin D effective radiation. By analogy with SPF we define an attenuation factor for previtamin D effective radiation AF_VD_ as

$${\text{AF}}_{\text{vd}}=\left(\int {\text{E}}_{\lambda }\;\left(\lambda \right)\;{\text{s}}_{\text{VD}}\;\left(\lambda \right)\;\text{d}\lambda \right)\;/\;\left(\int {\text{E}}_{\lambda }\;\left(\lambda \right)\;{\text{s}}_{\text{VD}}\;\left(\lambda \right)\;\text{T}\;\left(\lambda \right)\;\text{d}\lambda \right)$$

            (explanation of symbols see Introduction)

The estimated exposure time t_VD_ for getting an equivalent of the recommended vitamin D intake of 2000 IU/day is t_VD_ = 4 min x AF_VD_ when the protected skin is exposed to the above mentioned maximum solar input. The attenuation factors AF_VD_ and the exposure times t_VD_ for skin type 2 for the 4 types of sunscreens are given in Table 4.

**Table 4:** Attenuation factors AF_VD_ for previtamin D- effective radiation, exposure times (t_VD_) for skin type 2 needed per day for getting an equivalent of the recommended amount of 2000 IU vitamin D/day regarding 4 variations of sunscreens with SPF 6, 15 and 30, respectively.

Sunscreen Type	SPF	6	15	30
**1**	AFVD, tVD [min]	10.7, 42.8	40.2, 160.8	67.0, 268
**2**	AFVD, tVD [min]	7,8, 31.2	16.2, 64.8	34.0, 136
**3**	AFVD, tVD[min]	7.2, 28.8	15.4, 61.6	33.1, 132.4
**4**	AFVD, tVD[min]	6.1, 23.1	13.7, 52.2	30.6, 116.6

Table 4 includes the exposure times t_VD_ for getting the recommended equivalent of 2000 IU vitamin D/day. The exposure time t_VD_ for sunscreens e.g. with SPF = 30 are within the usual time frame of sun bathers.

**Fig 6:**
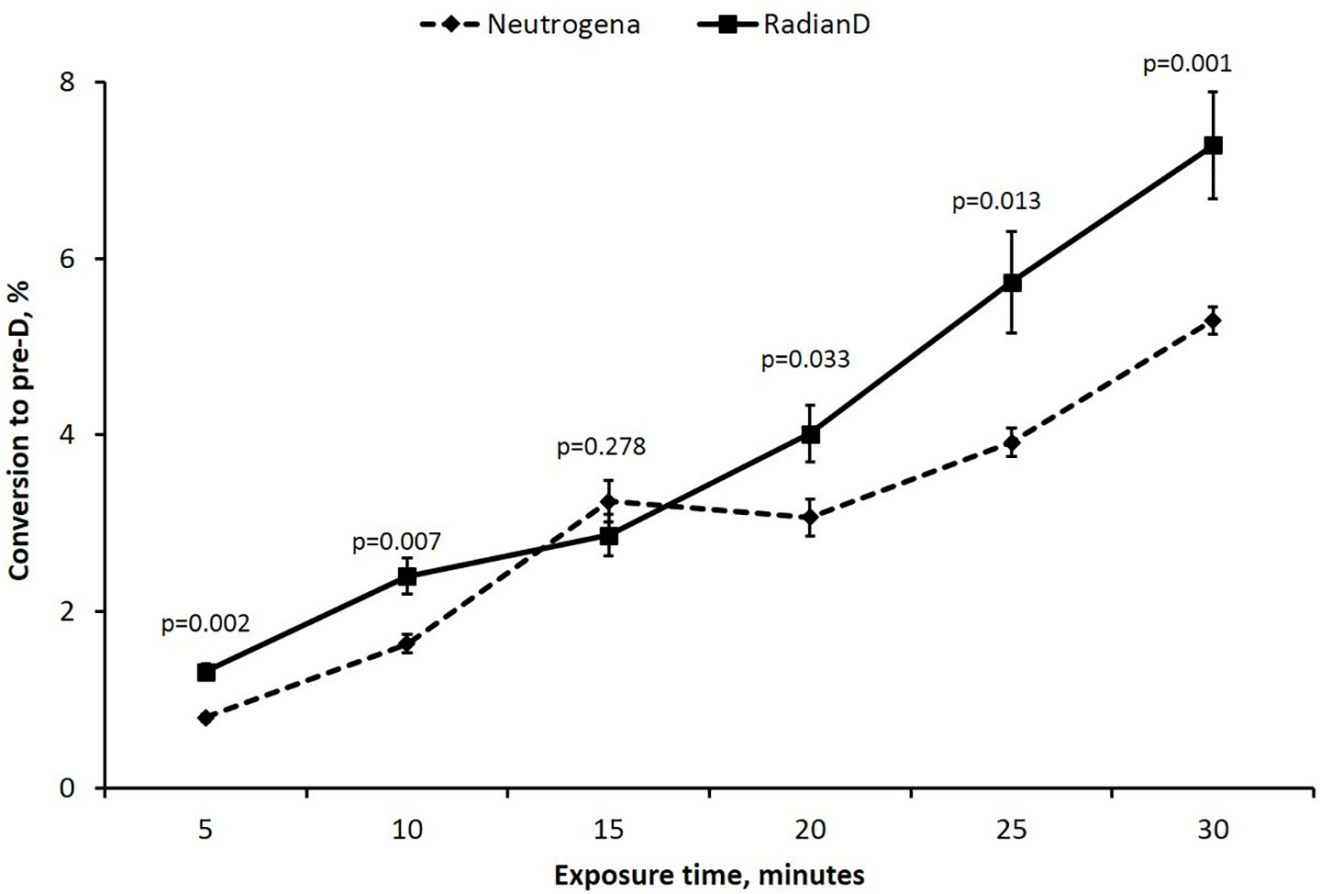
*In vitro* conversion of 7-DHC to previtamin D after irradiation with simulated UV solar radiation. Mean values +/- SE.

Results of *in vitro* experiments as described in Materials and Methods are shown in Fig 6.

A one-way analysis of variance was performed to test if the mean percent conversion to pre-vitamin D was different between the ampoules covered with SPF-15 Solar D and ampoules covered with SPF-15 Neutrogena at each of the 5 time points. Significance was set at P<0.05. Data were analyzed using SAS statistical software (version 9.3; SAS Institute Inc, Cary, NC).

This study demonstrated an increase of the previtamin D production for the experimental optimized sunscreen by a factor of approximately 1.5 compared to the commercial sunscreen.

Having all relevant data needed available (spectral transmittance of the UV absorbers used in both sunscreens, of the spectral irradiance of the fluorescent lamp [15], of the spectral transmittance of the borosilicate ampoule, of the spectral transmittance of the polyethylene food wrap), we ran the calculation. The results are listed in Table 5.

**Table 5:** Calculated SPF, E_VD_/E_er_ and AF_VD_ of both a commercially available sunscreen (Neutrogena) and an experimental sunscreen named Solar D which was optimized to a low attenuation factor AF_VD_.

Sunscreen	SPF (on label)	SPF (Calculated)	E_VD_/E_er_	AF_VD_
Neutrogena	15	17.6	1.06	55.9
Exp. sunscreen Solar D	15	12.3	2.08	24.2

The ratio of AF_VD_ regarding the two sunscreens is 55.9/24.2 = 2.3. Thus the calculated ratio is higher than the measured ratio of about 1.5. However, in any case a significant increase in previtamin D production was experimentally observed when using sunscreens optimized for previtamin D production.

During sun exposure the skin produces of vitamin D3. Vitamin D3 can either enter the circulation and be metabolized in the liver and kidney to 1,25(OH)2D3 or it can undergo a variety of metabolic steps in the skin producing several novel secosteroids that exert antiproliferative, pro-differentiation and anti-inflammatory effects on cultured skin cells.[16] Sun exposure is the major source of vitamin D for most children and adults worldwide. It is also recognized that vitamin D deficiency and insufficiency is a major health problem that afflicts approximately 40% and 60% of children and adults respectively. A major cause for this is the recommendation that no child or adult should ever be exposed to one direct ray of sunlight because of concern for increased risk for skin cancer. To avoid direct sun exposure widespread sunscreen use is implemented. However was not fully appreciated was that sunscreens are designed to efficiently absorbed radiation in the UVB range; an SPF of 30 absorbs 97.5%. The unintended consequence is that this radiation is also responsible for the cutaneous production of vitamin D. As a result an SPF of 30, properly applied, reduces the capacity of the skin to produce vitamin D by 97.5%. There are several chemical compounds that are typically used in a sunscreen that efficiently absorbed varying wavelengths of UVB radiation. In theory the judicious choice of compounds that have less UV absorbing properties at wavelengths near the peak action spectrum for previtamin D3 production i.e. 295 nm while retaining the ability to efficiently absorb the rest of the solar UVB radiation would provide a sunscreen that still had its anti-erythemal properties while permitting selective UVB radiation to be transmitted into the skin to produce previtamin D3. This manuscript describes a calculation method for optimizing a sunscreen for producing previtamin D3 and at the same time protecting against erythema. Solar D was designed with compounds with differing filter compositions to maximize previtamin D3 production while maintaining its sun protection for reducing erythema. When this sunscreen, that is rated as an SPF 15, was compared to a popular commercial sunscreen with the same SPF of 15, we observed *in vitro* a significant enhancement of as much as 50%as much as 50% in the production of previtamin D3 occurred with the solar D sunscreen compared to the popular commercial one. Based on our previous observations the *in vitro* results can be directly translated to what would be expected when the sunscreens are used on human skin. [4,5] Therefore we have proof of principal that a sunscreen can be produced for optimizing previtamin D3 production while retaining its sun protection factor for reducing erythema.
